# A rare case of coexistence of Wegener’s granulomatosis and pulmonary tuberculosis with subsequent development of thrombosis of the cerebral veins

**DOI:** 10.1186/s12879-021-06583-w

**Published:** 2021-09-14

**Authors:** Zohreh Rostami, Luca Cegolon, Nematollah Jonaidi Jafari, Nasrin Gholami, Seyed Vahid Mousavi, Fakhri Allahyari, Atena Azami, Mohammad Javanbakht

**Affiliations:** 1 Nephrology and Urology Research Center, Baqiyatallah University of Medical Sciences, Tehran, Iran; 2 Public Health Department, Local Health Unit N. 2 “Marca Trevigiana”, Treviso, Italy; 3 Health Research Center, Life Style Institute, Baqiyatallah University of Medical Sciences, Tehran, Iran; 4 Hematology Oncology Research Center, Tabriz University of Medical Sciences, Tabriz, Iran; 5 Atherosclerosis Research Center, Baqiyatallah University of Medical Sciences, Tehran, Iran; 6 Neuroscience Research Center, Baqiyatallah University of Medical Sciences, Tehran, Iran; 7 Department of Pathology, School of Medicine, Alborz University of Medical Sciences, Alborz, Iran

**Keywords:** Wegener’s Granulomatosis, Granulomatosis with polyangiitis, Tuberculosis, Thrombosis, Sigmoid and transvers sinus

## Abstract

**Background:**

Granulomatosis with polyangiitis (GPA), also known as Wegener’s granulomatosis, is an idiopathic systemic disease typically affecting the lungs, although other organs may also be involved.

**Case presentation:**

A 28-year-old male was admitted to Baqiyatallah university hospital in Teheran (Iran) after a 3-week history of fever and productive cough. The patient gradually developed fatigue, arthralgia, hematuria, nausea, vomiting, dyspnea, hemoptysis, weight loss, oliguria and then anuria. Chest-X-ray (CXR) and computerized tomography scan revealed cavitating nodular opacities in the right lung lobe. Furthermore, plasma creatinine increased from 2.2 to 4 mg/dl in a few days. Histopathological examination of kidney biopsy revealed peri-glomerular and peri-vascular inflammation, degeneration and necrosis of the tubular epithelial lining, red blood cell casts, distorted glomerular structure, fibrin thrombi, segmental breaks of the glomerular basal membrane, disruption of Bowman's capsular membrane and crescent formation of the affected glomeruli. An abnormal CXR, an abnormal urinary sediment and a typical kidney histology were used as criteria to diagnose glomerulonefritis with poliangiitis (GPA). Bronchoalveolar lavage smear and PCR turned out positive for mycobacterium tuberculosis. After 3 months of treatment for (GPA) and tuberculosis the patient developed headache and seizure. Cerebral Magnetic Resonance Venography revealed cerebral venous thrombosis of the sinus transverse and sigmoid.

**Conclusions:**

Tuberculosis may coexist with GPA, as it occurred in our patient. Since a crescentic glomerulonephritis can progress to renal failure, clinicians should always be aware of potential multiple conditions when considering differential diagnoses.

## Background

Granulomatosis with polyangiitis (GPA), also known as Wegener’s granulomatosis, is an idiopathic systemic disease which can affect multiple organs, particularly the upper and lower respiratory tract, the lung and the kidney [1–4]. GPA is a rare disease, with a prevalence estimated to be around 3/100,000 in the United States during 1979–1988 [5] and 134.9/million during 2013 in the United Kingdom, where the respective incidence was reportedly 11.8/million person-years during 1997–2013 [6].

GPA is featured by granulomatous inflammation, systemic vasculitis affecting medium and small arteries and necrotizing glomerulonephritis. Furthermore, GPA is characterized by cytoplasmic anti-neutrophil auto-antibodies (c-*ANCA*) [3, 7–9]. Because of the involvement of the respiratory system, some patients affected by GPA may be initially mis-diagnosed with tuberculosis (TB).

Whilst the health outcome of GPA patients is usually favorable if pharmaceutical therapy (relying on cyclophosphamide and methylprednisolone) is promptly started since the onset of the disease, the subsequent immune-suppression caused by the latter treatment regimen may increase the risk of infectious diseases [10–12].

Here, we describe the coexistence of a rare case of GPA and TB, with the subsequent onset of a cerebral venous thrombosis (CVT) of the sinus transverse and sigmoid following 3 months treatment for GPA as well as TB.

## Case presentation

A 28-year-old male presented at the accident & emergency (A&E) department of Baqiyatallah hospital in Tehran (Iran) with fever and productive cough for the past 3 weeks. Oral antibiotics (cefixime 400 mg/daily) had not been effective to treat his symptoms and the patient had progressively developed fatigue, arthralgia, hematuria, nausea, vomiting, dyspnea, hemoptysis, weight loss, oliguria and then anuria.

There was no relevant medical or family history for pulmonary disease (including TB) and the patient had no history of smoking or alcohol consumption.

At clinical examination blood pressure was 160/100 mmHg, respiratory rate 16 breaths per minute (BPM), heart rate 90 beats per minute (bpm), body temperature 38 °C and oxygen (O_2_) saturation was 93% in room air.

At admission White Blood Cells (WBC) were 19,300/μL, polymorphonuclears (PMN) 70%, Haemoglobin (Hb) 12 g/dl, MCV 85, platelets (PLT) 426 × 10^3^/μL, erythrocyte sedimentation rate (ESR) 114, C reactive protein (CRP) 91 mg/L, Blood Urea Nitrogen (BUN) 29 mg/dl, plasma creatine 2/2 mg/dl (rapidly increasing up to 6 mg/dl), plasma Na + 135 mEq/L and plasma K + 4.5 mEq/L.

During the oliguric stage, at urine analysis WBC were 3–5 × 10^3^/μL, proteins + 3 and no bacterial growth could be detected following 48 h urine culture. Furthermore, at serologic examination no antinuclear antibodies (ANA), no anti-double strand DNA (ds-DNA) nor anti-glomerular basal membrane (GBM) antibodies could be detected. Whilst serum levels of perinuclear anti-neutrophil cytoplasmic antibodies (p-ANCA), C3, C4, CH50 were all negative, c-ANCA were 2.2 U/ml (positivity threshold > 0.05 U/ml).

24-h urine proteins were 1,021 mg/day, anti-streptolysin O (ASO) titer was < 200 IU/ml and the level of angiotensin converting enzyme (ACE) was 11.5/µL.

**Fig. 1:**
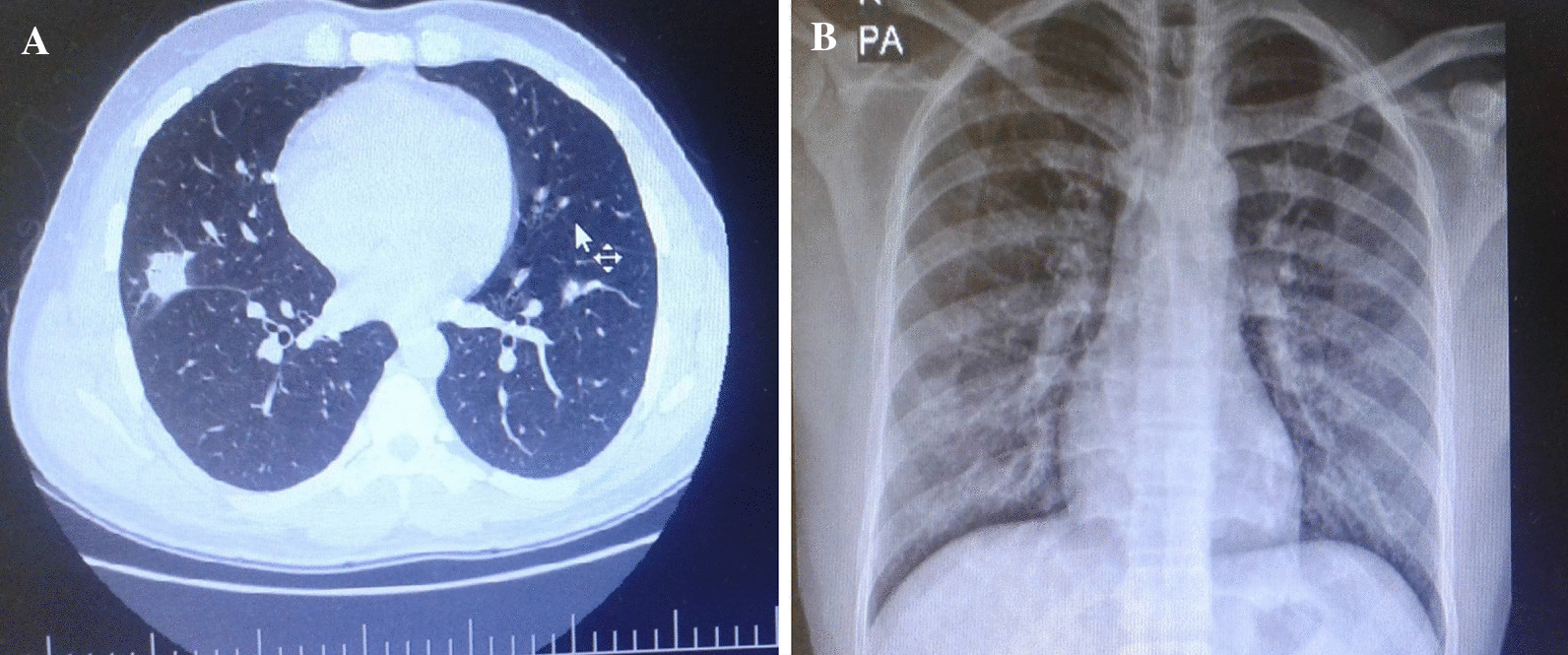
**a** Chest X ray. Opacity lesions visible in both lungs. **b** Chest spiral computerized tomography. Cavitary and nodular lesions visible in the right lower lung lobe

Chest X-ray (CXR) revealed opacity lesions (Fig. 1a) and spiral chest computerized tomography (CT) showed nodules in the right lung parenchyma (Fig. 1b). At ultrasonography (US), the echogenicity of the renal parenchyma was enhanced, with no signs of kidney stones or hydronephrosis.

An abnormal CXR, an abnormal urinary sediment and an indicative kidney biopsy were therefore reliable criteria to diagnose GPA in our patient, as recommended by the American College of Rheumatology [13, 14].

Although no endobronchial lesions could be observed at bronchoscopy and the purified protein derivative (PPD) skin test was negative, broncho-alveolar lavage (BAL) smear turned up positive for acid-fast bacilli, with TB being subsequently confirmed at PCR and (after 40 days) culture.

Since GPA and TB were diagnosed simultaneously and a crescentic glomerulonephritis is a life-threatening condition, a combined treatment against TB as well as GPA was promptly started at the same time, to avoid potential deterioration of patient’s health.

Treatment of GPA is divided into two stages [15]:

1. Induction stage; and
2. Remission maintenance stage.

The goal of induction therapy is to promptly inhibit the inflammation to control the disease and reduce a permanent tissue injury. In the remission maintenance stage, lower-dose immunosuppression is used to prevent relapses. Of course, achieving these goals should be pursued at minimal risk of toxicity.

Adjusted doses of intravenous cyclophosplamide combined with high-doses of intravenous steroids for first-line induction were started, as recommended for the severe forms of GPA [15]. Steroid therapy was continued with methylprednisolone (500 mg/daily i.v. pulse) for up to 3 days and maintained with 2 mg/kg/day oral prednisolone from day 4 onward, tapered to 15 mg/day prednisolone after 12 weeks and then gradually reduced to 5 mg/day. Cyclophosphamide (500 mg I.V. pulse) was administered every 2 weeks for 3 months, subsequently switched to azathioprine, which is less toxic as maintenance therapy once remission is achieved.

ANCA levels and other inflammatory markers such as ESR and CRP were monitored to assess the clinical condition of the patient and his response to therapy [16].

Standard therapeutic regimen of TB in renal disease was administered according to Center for Disease Control and Prevention (CDC) guidelines for patients on hemodialysis (creatinine clearance < 10 ml/min), adjusting the dose of pyrazinamide (25–35 mg/Kg, three times weekly after dialysis sessions). Dosage adjustment is mandatory for ethambutol, whose prolonged half-time in renal failure significantly increases the risk of retrobulbar optic neuritis [17, 18]. The dose of ethambutol was therefore adjusted (15–20 mg/Kg, three times weekly after dialysis sessions), but after several days had to be discontinued as our patient developed a blurred vision. Rifampicin (600 mg daily) and isoniazid (300 mg daily) were instead administered without renal dose adjustment, as recommended by CDC [19].

**Fig. 2:**
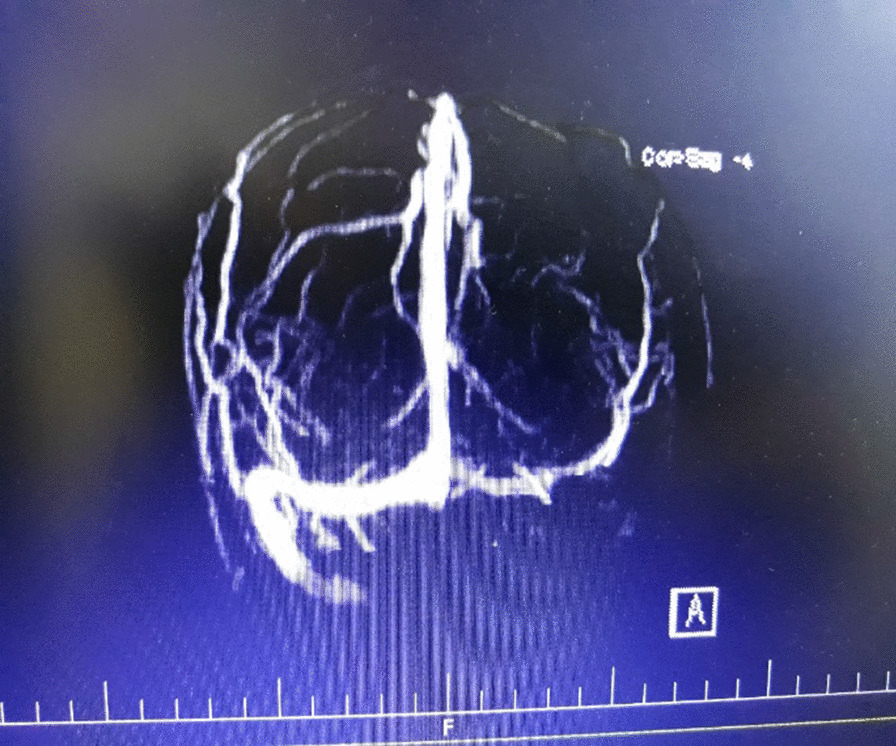
Brain magnetic resonance venography. A filling defect suggestive of thrombosis can be observed in the left transverse and sigmoid venous sinuses

Following 3 months of combined treatment for both TB and GPA, a novel onset of severe generalized headache non-responsive to analgesics and vomiting occurred and the patient developed seizures without fever over the course of 6 h. However, Magnetic Resonance Venography (MRV) revealed a CVT of the sinus transverse and sigmoid in the left side of the brain (Fig. 2). Protein S, protein C, antithrombin 3, Factor V Leiden, anticardiolipin antibodies, Beta-2 glycoprotein 1 antibody and lupus anticoagulant test were all within normal range.

Any form of infection (bacterial, fungal or viral) was excluded at smear, culture and PCR of the cerebrospinal fluid following lumbar puncture (LP).

**Fig. 3:**
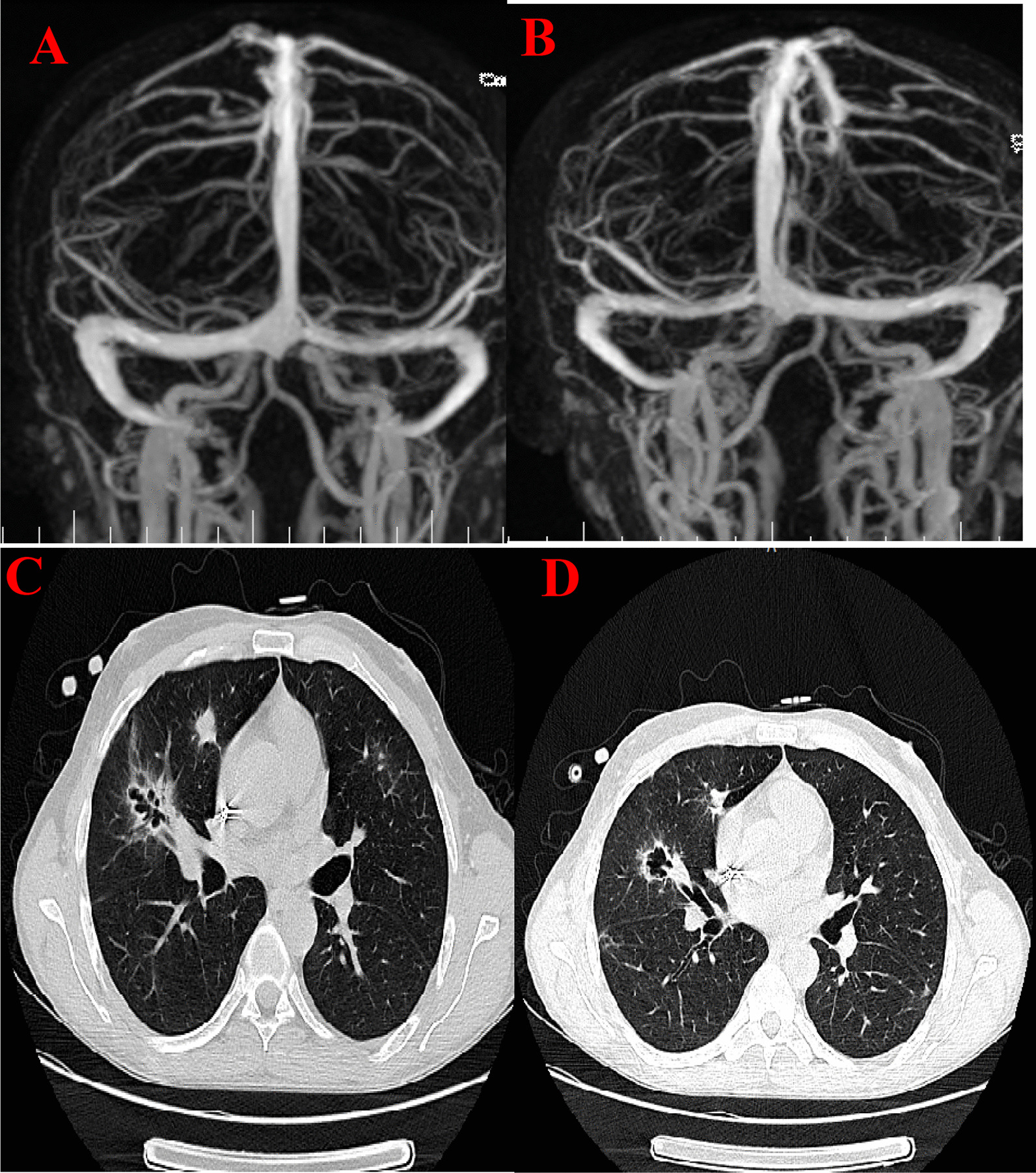
Magnetic resonance venography imaging after a 6 months treatment with warfarin (**A** and **B**): no evidence of thrombosis in the transverse and sigmoid sinus can be noted. CT image (**C** and **D**): multiple cavitary lesions and ground glass opacities in both lungs

After LP anti-coagulation with heparin and anticonvulsants was administered and all symptoms (including seizures and headache refractory to analgesics) dramatically improved. The patient was eventually discharged with a treatment scheme of oral warfarin. The goal of treatment was to achieve an international normalized ratio (INR) between 2.0 and 3.0 for at least 6 months. Neuro-imaging and CT were repeated at 6 months after presentation of thrombosis, which eventually resolved (as can be seen from Fig. 3).

**Fig. 4:**
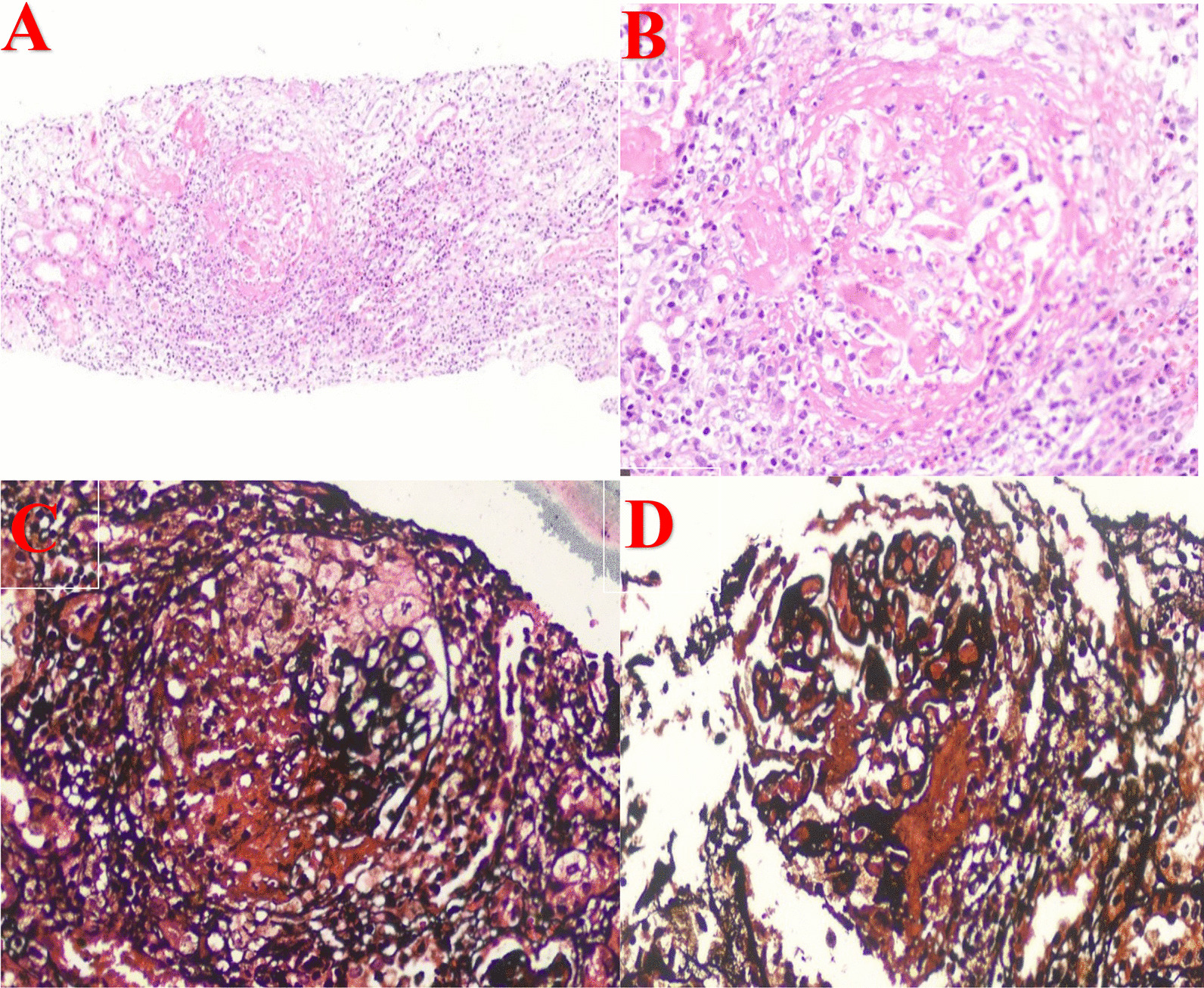
**A** Peri-glomerular and peri-vascular inflammation, degeneration and necrosis of the tubular epithelial lining. Some tubules contain red blood cell casts. **B** Distorted glomerular structure. Fibrin deposition in the capillary loops (fibrin thrombi) obliterating the capillary lumina, accompained by karyorrhectic debris in the glomerular tuft. Segmental breaks of the glomerular basal membrane (GBM and disruption of Bowman's capsular membrane are also visible. Renal parenchyma showing severe inflammatory response around an affected glomerulus. **C** Jones methenamine silver (JMS) stain shows an affected glomerulus with crescent formation consisting of proliferated parietal epithelial cells and fibrin between them. The crescent, obliterating Bowman's space and compressing the glomerular capillary tuft, is focally adherent to Bowman's membrane. A rupture of Bowman's capsular basal membrane is also visible. **D** An affected glomerulus showing large congestion with fibrin thrombi in the capillary loops and fibrin deposition in Bowman's space. Damage of the GBM and rupture of Bowman's capsule can also be noted

Kidney biopsy showed peri-glomerular and peri-vascular inflammation, degeneration and necrosis of tubules with red blood cell casts (Fig. 4A). Moreover, a distorted glomerular histology, with fibrin deposition in the capillary loops, segmental breaks of the glomerular basal membrane (GBM), disruption of Bowman's capsular membrane and a severe peri-glomerular inflammatory response could be observed (Fig. 4B). In addition, the affected glomeruli presented crescent formation of proliferated parietal epithelial cells with fibrin between them and a crescent obliterating Bowman's space and compressing the glomerular capillary tuft (Fig. 4C). Moreover, other histopathology findings indicated large congested glomeruli with fibrin thrombi and fibrin deposition in Bowman's space as well as damage of the GBM and rupture of Bowman's capsule (Fig. 4D)*.*

Once renal function resumed the patient was referred to hemodialysis. While still on hemodialysis three times weekly, following a 6 month combined therapy with prednisolone, azathioprine and warfarin, pulmonary symptoms, CXR and MRV abnormalities resolved and the patient was placed on a waiting list for kidney transplantation.

## Discussion and conclusion

Our patient was diagnosed with simultaneous GPA as well as TB. Any attempt to differentiate GPA from TB can be challenging, since the two conditions share similar clinical and radiological features [13, 20].

Detection of GPA relies on a blend of systemic semeiotics indicative of vasculitis, serology positive for c-ANCA and histopathology confirming crescentic glomerulonephritis, necrotizing vasculitis and granulomatous inflammation from a biopsy of relevant organ like skin, lung or kidney [1]. According to the American College of Rheumatology two or more of the following criteria are needed to diagnose GPA with a specificity of 92%: nasal/oral inflammation, abnormal urinary sediment, abnormal CXR and suggestive kidney histology [13, 21]. In our patient an abnormal CXR, an abnormal urinary sediment and a typical kidney histology were the criteria employed to diagnose GPA [13, 14].

ANCA can be used as diagnostic test for GPA and a marker of GPA activity [1]. Nevertheless, whilst featured by high specificity, sensitivity of ANCA is influenced by the stage and activity of GPA, being 91% vs. 63% for active vs. inactive disease [22] and 60% vs. 93% for local vs. systemic disease, respectively [1]. By contrast, the pooled specificty of ANCA was estimated to be 99% for active and 99.5% for inactive GPA [1]. Furthermore, the probability of false positive results with ANCA serology is inversely related with the prevalence of GPA in the general population [1].

In a case report from India, a 50-year-old female presumed to be affected by TB based upon her clinical and radiological features, was eventually diagnosed with GPA following detection of ANCA and vasculitis, while she had already been placed on TB treatment [23]. Our patient was also positive for c-ANCA, which can also be found in TB and other respiratory diseases though [24], leading to potential misdiagnosis and improper management of GPA.

Since c-ANCA levels can also be elevated in TB patients and response of c-ANCA to anti-TB treatment can be rather variable, diagnosing a concomitant GPA in a TB patient can be challenging [25, 26]. That is why a biopsy is highly recommended for a diagnostic confirmation in such clinical circumstances [13].

Glomerulonephritis—especially pauci-immune necrotizing crescentic glomerulonephritis with necrotizing vasculitis—associated with TB infection is extremely rare. Albeit it may develop following immunosuppressive therapies, TB was coexisting with GPA in our patient, whose CXR showed radiological pulmonary opacities and a chest CT scan showed unilateral cavitations and multiple nodules in the right lung parenchyma. Based on the latter radiological pattern and considering the patient’s clinical history, TB was suspected and eventually confirmed by BAL smear and PCR.

Three different mechanisms of rapidly progressive glomerulonephritis have been recognized, with 40% cases estimated to be caused by granular deposition of immune complexes in the glomeruli, 10% caused by linear deposition of IgG anti-GBM on the GBM, and a third pauci-immune form featured by cellular crescents and necrotizing areas without granulomas [27–29].

A crescentic glomerulonephritis could also exceptionally be the result of pulmonary TB, which can determine nephrotic syndrome and progressive renal failure [30]. The kidney can be impaired by TB in multiple fashions, the most common of which is direct infection of the urinary tract by Mycobacterium tuberculosis. Further renal conditions potentially arising from TB comprise chronic interstitial nephritis and amyloidosis [4]. Renal TB is typically featured by epithelioid granulomas, with or without caseation, frequently including Langhans-type giant cells. However, whilst TB related glomerulonephritis is typically characterized by immune complex deposition, our patient had a pauci immune form.

Prednisolone and cyclophosphamide are the gold standard to treat severe forms of GPA. Since GPA can be ANCA negative in 10% cases, immunosuppressive treatment should be started as soon as possible in highly suspicious GPA, irrespective of auto-antobody serology [31] Immunosuppressive treatment is in fact critical to decrease morbidity and mortality in the long term [14]. Although the latter treatment regimen allows remission in over 90% of GPA patients, the kidney function should be consistently verified before treatment start [13]. As our patient had a compromised renal function, TB treatment was adjusted based upon creatinine clearance, as recommended by CDC [19].

Despite our patient survived, his kidney function could not recover and hemodialysis had to be continued. The patient was then re-admitted to hospital 3 months following discharge, for the onset of headache and tonic–clonic seizures associated with CVT.

CVT can be idiopathic or caused by infectious and non-infectious factors [32], including inflammatory diseases (such as GPA, systemic lupus erythematosus, Behçet’s disease, inflammatory bowel disease, sarcoidosis), head traumas involving the venous sinuses, infections of the head and neck district, iron deficiency anaemia and haematological conditions (myeloproliferative disorders associated with JAK2 V617F mutations, paroxysmal nocturnal haemoglobinuria and haemoglobinopathies) [32–35]. Patients can be also affected by multiple risk factors for CVT [35].

Albeit thrombosis of the superior sagittal veins, extremities deep veins, pulmonary veins, portal veins and hepatic veins have been reported to be potentially associated with TB [32, 36–38], local infection is needed to trigger the thrombotic process. Since direct smear, culture and PCR of the cerebrospinal fluid were all negative for bacterial, fungal or viral infection, CVT was likely caused by GPA in our patient. Following diagnosis of CVT, the administration of heparin effectively quenched seizures and an headache non-responsive to analgesics in our patient, who was eventually discharged on oral warfarin therapy.

In conclusion, this is the first case report presenting a coexisting diagnosis of GPA and TB, with subsequent onset of CVT - likely caused by GPA and/or its respective therapy - 3 months after the start of treatment for the latter two diseases. Clinicians should therefore always be aware of potential multiple conditions when considering differential diagnoses. Whilst immunosuppressive therapy is relatively contraindicated with active TB, untreated GPA might be life threatening and would result in unfavourable renal outcome in the long term. Moreover, combined treatments for both GPA and TB showed positive patient response, according to published case reports [29, 39, 40]. Patient's symptoms attributable to CVT (seizures and an headache refractory to analgesics) were effectively controlled with heparin, followed by a domiciliary oral warfarin scheme post-discharge.

## Data Availability

All data discussed in the manuscript are included within this published article.

## Abbreviations

BAL: Bronchoalveolar lavage
CT: Computerized tomography
CVT: Cerebral venous thrombosis
GBM: Glomerular basal membrane
GPA: Granulomatosis with polyangiitis
MRV: Magnetic resonance venography
PPD: Purified protein derivative
TB: Tuberculosis
US: Ultrasonography
